# Mitophagy in Cardiovascular Diseases

**DOI:** 10.3390/jcm9030892

**Published:** 2020-03-24

**Authors:** Giampaolo Morciano, Simone Patergnani, Massimo Bonora, Gaia Pedriali, Anna Tarocco, Esmaa Bouhamida, Saverio Marchi, Gina Ancora, Gabriele Anania, Mariusz R. Wieckowski, Carlotta Giorgi, Paolo Pinton

**Affiliations:** 1 Maria Cecilia Hospital, GVM Care & Research, Via Corriera 1, Cotignola, 48033 Ravenna, Italy; mrcgpl@unife.it (G.M.); simone.patergnani@unife.it (S.P.); pdrgai@unife.it (G.P.); 2 Department of Medical Sciences, Laboratory for Technologies of Advanced Therapies (LTTA), University of Ferrara, 44121 Ferrara, Italy; bnrmsm1@unife.it (M.B.); trcnna@unife.it (A.T.); esmaa.bouhamida@unife.it (E.B.); grgclt@unife.it (C.G.); 3 Neonatal Intensive Care Unit, University Hospital S. Anna Ferrara, 44121 Ferrara, Italy; 4 Department of Clinical and Molecular Sciences, Marche Polytechnic University, 60126 Ancona, Italy; s.marchi@staff.univpm.it; 5 Neonatal Intensive Care Unit, Infermi Hospital Rimini, 47923 Rimini, Italy; gina.ancora@auslromagna.it; 6 Department of Medical Sciences, Section of General and Thoracic Surgery, University of Ferrara, 44121 Ferrara, Italy; gabriele.anania@unife.it; 7 Laboratory of Mitochondrial Biology and Metabolism, Nencki Institute of Experimental Biology of the Polish Academy of Sciences, 3 Pasteur Str., 02-093 Warsaw, Poland; m.wieckowski@nencki.edu.pl

**Keywords:** mitophagy, cardiovascular diseases, mitochondria, autophagy

## Abstract

Cardiovascular diseases are one of the leading causes of death. Increasing evidence has shown that pharmacological or genetic targeting of mitochondria can ameliorate each stage of these pathologies, which are strongly associated with mitochondrial dysfunction. Removal of inefficient and dysfunctional mitochondria through the process of mitophagy has been reported to be essential for meeting the energetic requirements and maintaining the biochemical homeostasis of cells. This process is useful for counteracting the negative phenotypic changes that occur during cardiovascular diseases, and understanding the molecular players involved might be crucial for the development of potential therapies. Here, we summarize the current knowledge on mitophagy (and autophagy) mechanisms in the context of heart disease with an important focus on atherosclerosis, ischemic heart disease, cardiomyopathies, heart failure, hypertension, arrhythmia, congenital heart disease and peripheral vascular disease. We aim to provide a complete background on the mechanisms of action of this mitochondrial quality control process in cardiology and in cardiac surgery by also reviewing studies on the use of known compounds able to modulate mitophagy for cardioprotective purposes.

## 1. Fundamental Aspects of Mitophagy

Recent decades have been characterized by robust success in efforts to reduce mortality due to heart disease; however, heart disease remains one of the leading causes of death. Every year, approximately 18 million people around the globe die from cardiovascular diseases (CVDs) [1]. Advances in clinical care and the identification of novel pathogenetic mechanisms and new drug targets are necessary to reduce the acutely lethal manifestations of heart disease. Several areas of challenge have emerged as possible platforms for designing innovative therapeutic approaches. For example, pathways involving mitochondria play major roles in heart physiology [2]. Indeed, mitochondria constitute approximately 30–40% of the cardiomyocyte volume [3], and cardiomyocyte function is closely associated with mitochondrial status; thus, CVDs have unsurprisingly been linked to mitochondrial dysfunction [4,5,6].

Mitochondria are considered the “energy powerhouses of cells”. Within these organelles, a large proportion of cellular adenosine triphosphate (ATP) is generated from the degradation of sugars and long-chain fatty acids (FAs) and from the metabolism of amino acids and lipids. Cardiac mitochondria use 60% to 90% of the energy originating from FA oxidation (FAO) as their primary energy source (they also use glucose, pyruvate, and lactate) to improve cardiac function, whereas most other organs use glucose as the major energy substrate [7]. Mitochondria are deeply involved in calcium (Ca^2+^) handling [8,9,10]. Recent advances in the identification of the molecular partners regulating Ca^2+^ dynamics in the mitochondrial compartment have helped elucidate the critical contributions of mitochondria to (patho) physiological conditions that rely on mitochondrial Ca^2+^ oscillations [11]. Mitochondria are also the primary sources of intracellular reactive oxygen species (ROS) [12]. These dangerous species are produced in the electron transport chain (ETC) due to the partial reduction of oxygen to superoxide by complex I and complex III and are very harmful to cells. Accordingly, ROS activate several cell death pathways, including necrosis and apoptosis pathways; more importantly, they also cause severe damage to DNA [13]. Mitochondria possess their own DNA (mtDNA), which is almost exclusively maternally inherited [14,15] and consists of base pairs that constitute 37 genes encoding 13 polypeptides essential for oxidative phosphorylation (OXPHOS) plus 22 tRNAs and two rRNAs. Unlike nuclear DNA (nDNA), mtDNA has rudimentary DNA repair mechanisms and is not protected by histones. Hence, mtDNA is more susceptible to ROS-induced damage than nDNA. Indeed, the mutation rate of mtDNA has been estimated to be approximately 15-fold higher than that of nDNA [16].

Clearly, these elements highlight the importance of preserving mitochondrial integrity. Mitochondrial dysfunction can impact various aspects of normal cell life and ultimately induce severe alterations that can lead to the death of the cell itself. Mitochondria have developed two different mechanisms to maintain a healthy status and guarantee an active quality control system. In the first mechanism, biogenesis, fission and fusion cooperate with each other to increase the mitochondrial population under conditions of high energetic demand or to permit a damaged organelle to fuse with a healthy mitochondrion and replace its damaged or lost components [17,18]. In brief, biogenesis is regulated by a series of transcription factors like Transcription Factor A, Mitochondrial (TFAM), Transcription Factor B2, Mitochondrial (TFB2M), Nuclear Respiratory Factor 1 (NRF1), Nuclear Factor, Erythroid 2 Like 2 (NRF2), Estrogen Receptor-related Receptor-alpha (ERRs) and Peroxisome proliferator-activated receptor Gamma Coactivator 1-alpha (PGC-1α) that ensure the maintenance of mitochondrial mass and positively regulate other genes involved in OXPHOS, heme biosynthesis and the import of proteins into mitochondria. Instead, fission and fusion rely on a pool of proteins mainly localized in mitochondria, such as mitofusin (MFN) 1 and 2 in the outer mitochondrial membrane (OMM) and optic atrophy 1 (OPA1) in the inner mitochondrial membrane (IMM), which are needed for the fusion of mitochondrial membranes and mtDNA exchange, and dynamin-related protein 1 (DRP1) and fission mitochondrial 1 (FIS1), which allow mitochondrial division, especially for the transmission of mitochondria to daughter cells in mitosis. In healthy cells, the mitochondrial network is highly interconnected, and there is a balance between the two mechanisms: impaired fusion leads to mitochondrial fragmentation, and unbalanced fission leads to mitochondrial elongation.

**Figure 1:**
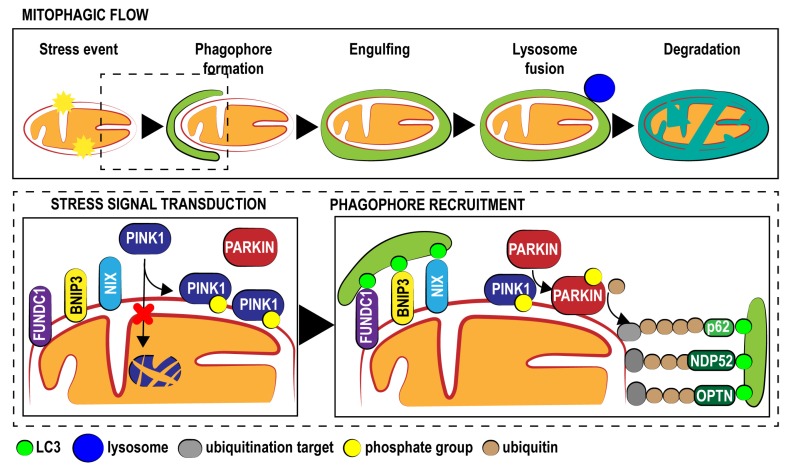
Molecular routes of mitophagy. Schematic representation of mitochondrial fate during mitophagy (upper panel). Representation of rearrangements of effectors onto the OMM in response to stress stimuli (lower left panel) and participants in the recruitment of the phogophore around the OMM (lower right panel) during mitophagy. OMM, outer mitochondrial membrane; FUNDC1, FUN14 Domain Containing 1; BNIP3, BCL2/adenovirus E1B 19 kDa protein-interacting protein 3; NIX, NIP-3-Like Protein X; PINK1, PTEN-induced kinase 1; PARKIN, Parkin RBR E3 Ubiquitin Protein Ligase; NDP52, Nuclear Domain 10 Protein 52; OPTN, Optineurin; LC3, Microtubule Associated Protein 1 Light Chain 3.

In contrast, the second mechanism is expected to remove damaged organelles. This mainly occurs through a cellular mechanism referred to as mitochondrial autophagy (mitophagy, Figure 1) [19].

First described over 50 years ago, autophagy is responsible for the sequestration and degradation of cytosolic components via the lysosomal pathway. During this conserved and physiologic process, a double-membrane vesicle (autophagosome) engulfs cellular material and then fuses with a lysosome to degrade it [20]. When discovered, autophagy was thought to be a nonspecific process in which cytosolic material was randomly sequestered. Autophagy has also been described as a selective mechanism capable of targeting viruses and bacteria (xenophagy), portions of the endoplasmic reticulum (ER; reticulophagy), peroxisomes (pexophagy) and mitochondria (mitophagy) [21].

Among the types of autophagy, mitophagy has become the most described and studied. Several conditions (cellular differentiation, fertilization, oxygen deprivation) and molecules (drugs and proteins) modulate this process. Furthermore, variations in the levels of mitophagy have been found in a diverse array of human pathologies, including cancer, neurodegeneration and CVDs [22].

Mitophagy was first observed in yeast, when two independent research groups screened for mitophagy-deficient mutants and identified the autophagy-related gene 32 (Atg32), which is essential for mitophagy. Previous studies have suggested that the gene products of Aup1p and Uth1p are involved in the autophagic degradation of mitochondria [23,24]; however, these factors were not identified during genomic screening. In mammals, the first observation of mitophagy was made during the maturation of reticulocytes. Mature red blood cells are devoid of mitochondria because immature red blood cells lose their mitochondria via mitophagy during differentiation. This event is mediated by the OMM protein BCL2/Adenovirus E1B 19 KDa Protein-Interacting Protein 3-Like (BNIP3L; also known as NIP-3-Like Protein X, NIX), whose expression increases during maturation. NIX contains a motif required for binding to microtubule-associated protein 1 (MAP1) light chain 3 (LC3) [25,26], which is localized on the surface of the autophagosomal membrane and mediates the sequestration of mitochondria into autophagic vesicles. Subsequent studies have found that Nix/BNIP3L shares 50% homology with BCL2 Interacting Protein 3 (BNIP3) [27]. Interestingly, this factor is involved in autophagy and mitophagy under hypoxic conditions both in cancer and during myocardial ischemia/reperfusion (I/R) injury (IRI) [28,29]. These findings suggest that mitophagy in mammals may be a mechanism conserved across various cell types and not only in reticulocytes. Accordingly, mitophagic elements have been unveiled in all human tissues, and a specific molecular mechanism governing mitophagy has been found. The discovery of this cellular pathway arose from research on Parkinson’s disease [30,31], which revealed a particular form of recessive Parkinsonism characterized by mutation in two genes: Parkin RBR E3 Ubiquitin Protein Ligase (PARK2), which codes for a cytosolic E3 ubiquitin ligase named Parkin, and PTEN-induced kinase 1 (PINK1), whose protein product is a kinase localized on the mitochondrial surface [32,33].

Normally, PINK1 enters mitochondria through the activity of translocase of the outer membrane (TOM) and reaches the mitochondrial inner membrane through the activity of translocase of the inner membrane (TIM), which recognizes an amino-terminal mitochondrial targeting sequence. During these importing steps, PINK1 is subjected to a series of proteolytic cleavage events by the intermembrane serine protease presenilin-associated rhomboid-like protein (PARL), and the full-length form of 64 kDa is sequentially cleaved into fragments of 60 kDa and 52 kDa [34,35]. Once released in the cytosol, the 52-kDa fragment is then degraded by the proteasome [36]. Overall, these mechanisms permit the maintenance of very low levels of PINK1 during unstressed conditions.

When a mitochondrial population becomes damaged, the mitochondrial import of full-length PINK1 throughout the TIM/TOM complex is disrupted; PINK1 then accumulates on the OMMs of only injured mitochondria and is stabilized in a complex composed of TOM7, TOM40, TOM70, TOM20 and TOM22 [37,38]. PINK1 mediates two different phosphorylation events aimed at converting the autoinhibited E3-ubiquitin (Ub) ligase Parkin into an active phospho-Ub-dependent enzyme. The first step involves autophosphorylation at S402, S228 and T257. Interestingly, mutations in these residues abolish both PINK1 activity and Parkin recruitment at the mitochondrial surface [39]. The second event is the direct phosphorylation of Parkin at S65 in the N-terminal Ubl domain, which increases its E3 ligase activity [40]. Finally, PINK1 provokes the addition of a phosphate onto S65 of Ub. As a result of this complex regulatory mechanism, the E3 ligase activity of Parkin is activated, allowing it to ubiquitinate mitochondrial proteins via direct interaction with phospho-Ub conjugates on the mitochondria. The presence of these poly-Ub chains promotes the recruitment of specific Ub-binding autophagy receptors to connect damaged mitochondria to LC3-positive phagosomes for clearance in lysosomes. Initial studies suggested that p62/sequestosome 1 (p62/SQSTM1) was the main receptor involved in mitophagy [41]. Subsequent studies have demonstrated that in addition to p62, at least four other receptors, neighbor of Brca1 (NBR1), nuclear dot protein 52 (NDP52), optineurin (OPTN) and TAX1BP1 (TBK1), are involved during the selective removal of damaged mitochondria. Despite the large number of studies, which adaptor is effectively essential for mitophagy remains unclear [42]. Experiments performed on cell lines with penta-knockout (KO) for all five receptors have suggested that only Nuclear Domain 10 Protein 52 (NDP52) and OPTN are effectively required for mitophagy. However, these experiments were performed on a single cell line, and not all cell types and tissues have comparable levels of these molecules [43].

## 2. Mitophagy in Cardiovascular Diseases

### 2.1. Atherosclerosis

**Figure 2:**
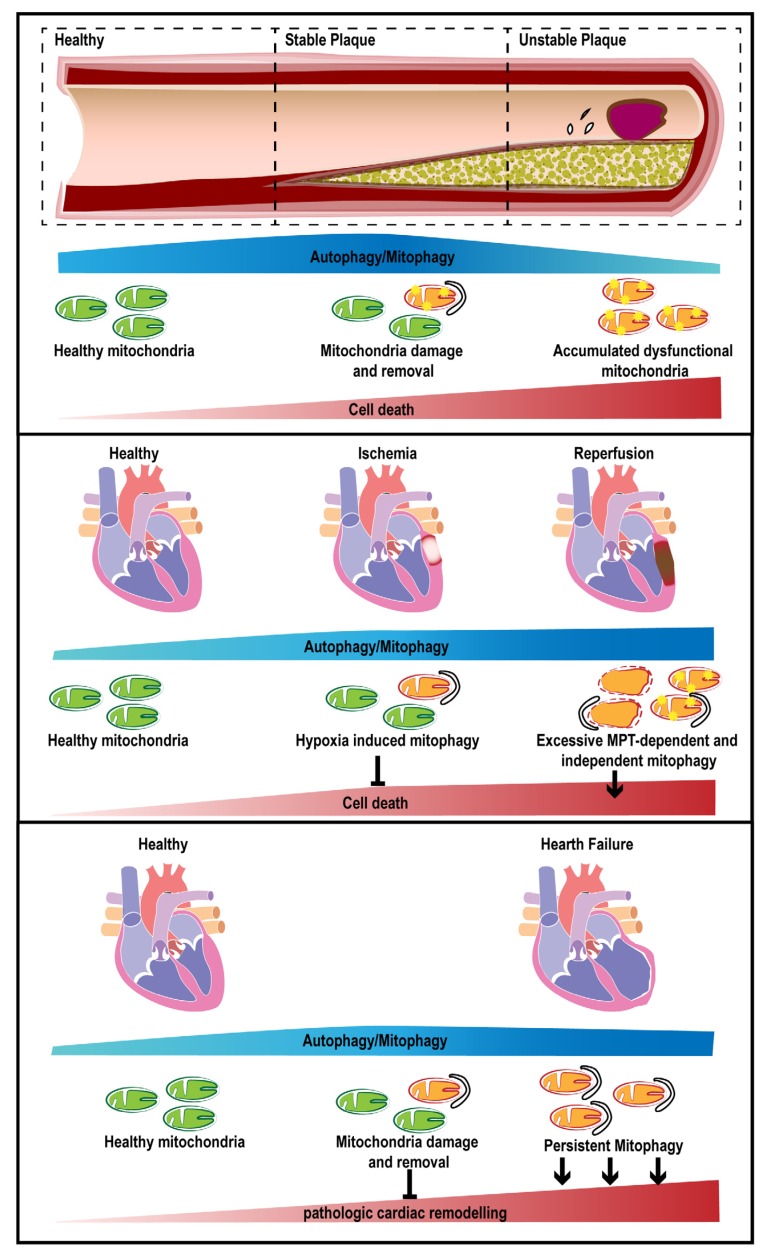
Involvement of mitophagy in major CVDs. Schematic representation of tissue remodeling during atherosclerosis (AS) (upper panel), I/R (middle panel) and heart failure (HF; lower panel) with related alterations in mitophagy extent, cell death or tissue remodeling. CVDs, cardiovascular diseases; I/R, ischemia/reperfusion; MPT, mitochondrial permeability transition; HF, heart failure.

Atherosclerosis (AS) is a chronic inflammatory disease that is very prevalent in industrialized nations. The interplay among lipid accumulation, increased vascular smooth muscle cell (VSMC) proliferation, matrix turnover, calcification and inflammation cause a significant narrowing of the arteries because of plaque buildup inside the artery lumen. In the last 10 years, various methods have been applied to assess autophagy in cells that form atherosclerotic plaques (VSMCs, endothelial cells, macrophages), including electron microscopy, fluorescence microscopy and western blot approaches [44,45]. Studies conducted on human specimens and mouse models of AS have reported either dysfunctional or decreased autophagy based on detection of the autophagic markers p62 and LC3-II in cells isolated from plaques [46,47,48]. Indeed, a strong decrease in LC3-II expression has been reported in patients with unstable plaques compared to those with stable plaques [48]; such decreases would enable dead cell accumulation in the artery wall and subsequent plaque destabilization (Figure 2). A dysfunction in the autophagic process, caused by either autophagy related 7 (ATG7) deletion in VSMCs [49,50] or a macrophage-specific ATG5-null mutant mouse model [46], has been linked to atheroma development in AS. In the latter, cholesterol crystals are not removed from plaques and trigger an increased secretion of interleukin (IL)-1β after macrophage inflammasome hyperactivation [46]. In humans and animals, complete loss of autophagy (like ATG5-null cells) is incompatible with life, and according to some authors, a slight decrease in autophagy is not always associated with experimental atherosclerosis [46]; therefore, further severe dysfunctions would be expected to occur in this pathway. One of these could be progressive lysosomal impairment with a concomitant accumulation of p62, a protein responsible for bringing polyubiquitinated proteins to the autophagosome for lysosome-dependent degradation.

Much less is known about mitophagy in the context of AS than about mitophagy in a general sense. Overall, mitophagy, together with fission and fusion dynamics, helps clear damaged mitochondria by optimizing the function of the total mitochondrial population inside cells [51]. Given that ROS and inflammation play pivotal roles in the progression of disease, activation of mitophagy by oxidized low-density lipoprotein (ox-LDL, an atherosclerotic agent) [52] and through exogenous melatonin administration [53] counteracts AS progression by stabilizing atherosclerotic plaques (Table 1). Indeed, melatonin was able to modulate mitophagy via a SIRT3/FOXO3a/Parkin-dependent signaling pathway and to attenuate IL-1β secretion.

### 2.2. Ischemic Heart Disease

A consequence of AS is ischemic heart disease (IHD), where the full or partial recovery of myocardial function is always difficult to achieve due to severe mitochondrial impairment [75,76]. Events consisting of I/R phases are accompanied by oxidative stress, pH changes, and cytosolic and mitochondrial Ca^2+^ overload, which lead to the opening of the mitochondrial permeability transition pore complex (PTPC) and contribute to cardiomyocyte death [12,77,78,79,80].

Autophagy (which is activated by increased cytosolic Ca^2+^ concentrations [81]) and mitophagy are known to be features of I/R-related diseases, and ex vivo experiments on cardiac ischemia, such as those performed with the Langendorff system, have made great contributions to these findings. Lysosomal alterations were first observed in perfused rabbit hearts in 1980; after 40 min of ischemia, the number of autophagosomes increases and peaks during reperfusion, suggesting that both ischemia and reperfusion can induce autophagy [82]. These findings have also been replicated in the mouse heart in vivo [83]. I/R-dependent upregulation of autophagy has been assessed by the detection of increased LC3-II and Beclin1 protein expression in association with Bcl-2-associated athanogene (BAG-1), a multifunctional prosurvival molecule [84]. Enhanced autophagy has also been detected after hypoxia treatment combined with glucose deprivation and subsequent reperfusion in fetal mouse hearts; localized lysosomal digestion limits injury to the entire cell, suggesting a role for the autophagic process in the repair of sublethal injury [85]. Thus, autophagy appears to be an important mechanism in cardiac tissue protection during and after hypoxia; indeed, inhibition of autophagy in animals through wortmannin treatment impairs the induction of LC3-II formation and Beclin1 overexpression, limiting cardioprotection [84].

In cultured cardiomyocytes, ischemia and glucose deprivation cause significant reductions in the levels of ATP that coincide with the upregulation of adenosine monophosphate-activated protein kinase (AMPK)-dependent autophagy and the inhibition of the mammalian target of rapamycin (mTOR) pathway. Accordingly, transgenic mice overexpressing dominant-negative AMPK display a diminished induction of autophagy following ischemia [83]. At the time of reperfusion, when physiological conditions are restored, AMPK is no longer activated; thus, considering previous findings, autophagy likely proceeds through an AMPK-independent mechanism, and some data have suggested that it occurs via Beclin1 overexpression [83].

Statistically, autophagy and mitophagy may be classified as protective mechanisms, but this could be an oversimplification. Under conditions in which the heart experiences energy loss, such as ischemia, the autophagic pathway may be protective, as it preserves basal metabolic requirements; however, induction of autophagy under other circumstances, such as at the time of reperfusion, may be detrimental. In agreement with this hypothesis, inhibition of Beclin1 has been reported to protect against myocyte death in vivo [83]. Other evidence has come from the use of urocortin, an endogenous cardiac peptide previously shown to reduce other forms of myocyte cell death induced by I/R; urocortin downregulates Beclin1 expression by activating the phosphoinositide 3 (PI3) kinase/Akt pathway [86].

Reperfusion of the heart is well accepted to lead to the opening of the PTPC [87,88]; however, the link between the PTPC and autophagy (or mitophagy) activation is poorly understood, and the literature remains controversial. As PTPC opening leads to mitochondrial membrane potential loss and mitochondria depolarization, the mitophagic pathway is expected to be activated. Indeed, mitophagic pathway activation is ascribed to PTPC opening in hepatocytes of rats and mice and in livers of human patients [89,90], where PTPC opening also plays a role in starvation-induced mitophagy [91]. Accordingly, blockade of pore opening with the known inhibitor cyclosporine A (CsA) has been found to prevent both mitochondrial swelling and autophagy caused by calpain 10 overexpression [92]. Moreover, overexpression of PINK1 in cardiac cells protects the cells from death by significantly delaying the onset of PTPC opening [93]; if PINK1 is depleted, as is the case in KO animals, the heart becomes increasingly vulnerable to ex vivo IRI. Between PTPC activity and autophagy/mitophagy, a feedback loop may exist where each pathway, in turn, controls the other one. PTPC opening can give rise to an autophagic-dependent cell repair mechanism when few mitochondria are involved during a moderate insult, but this process may become inadequate under extensive damage where cells tend to die.

On the other hand, a study headed by Gustafsson AB suggested that BNIP3-dependent mitophagy (induced upon hypoxia and in failing hearts [94,95]) in adult cardiomyocytes does not depend on PTPC opening [96] but only requires BNIP3 and constitutively expressed Beclin1 and autophagy related 5 (ATG5) [97].

Additionally, ER stress and the unfolded protein response (UPR) have been associated with the induction of autophagy and mitophagy [54,98,99]. Studies performed on both cultured neonatal rat and adult mouse ventricular myocytes have shown activation of ER stress and UPR processes under I/R conditions [100,101,102]. Indeed, hypoxia activates the UPR in surviving myocytes in the border zones of infarcted hearts [103]. Furthermore, activation of the ER stress response was found to be involved in the development of IHD in a predisposed transgenic murine model [104].

I/R reportedly induces excessive mitochondrial fission; this pathway can lead to the generation of two mitochondrial populations with either increased or decreased membrane potential. Most of the time, the depolarized population is not able to fuse and undergoes mitophagy-dependent removal. Thus, mitophagy is dependent on mitochondrial fission; inhibition of the dynamic process through the expression of dominant-negative Drp1^K38A^ or through Fis1 RNA interference results in disruption of mitophagy and subsequent accumulation of dysfunctional mitochondria [105]. Before Drp1 moves to mitochondria to allow fission, “marked” organelles are subjected to early-stage constriction at the mitochondria-associated membranes (MAMs), highlighting an active role for these microdomains in the choice of division sites, the recruitment of proteins and adaptors and the fulfillment of that process [18,106,107]. Further evidence derives from the study of inverted formin 2 (INF2), an ER-localized protein that orchestrates upstream Drp1, actin filament rearrangement and calcium fluxes at MAMs from the ER to mitochondria, which are essential to guarantee mitochondrial fission [108,109].

Other evidence of the cardioprotective roles of mitophagy has been described; for example, Fun14 domain-containing protein 1 (FUNDC1), a mitophagy receptor that interacts with LC3, is able to mediate the process in response to hypoxia [55,57] and has been found to regulate mitochondrial homeostasis, protecting the heart from IRI, in a mouse model [56]. Indeed, FUNDC1 loss of function through casein kinase 2α (CK2α)-mediated phosphorylation leads to inhibition of mitophagy and significant enhancement of tissue damage [110].

During in vivo acute myocardial infarction (MI) induced by permanent ligation of the left coronary artery in transgenic mice, autophagy is enhanced to preserve cellular ATP levels and protect cardiomyocytes from ischemic death [111] (Figure 2).

High-mobility group box-1 protein (HMGB1) is able to migrate to mitochondria and bind with mitophagy-related proteins after exogenous administration. In vivo evidence has shown that HMGB1 treatment in a murine model of acute MI might induce cardiomyocyte survival, protecting infarcted hearts [58]. Attenuation of apoptosis has been ascribed to the induced activation of AMPK and inhibition of mTOR complex 1 (mTORC1) [58]. Experiments on transgenic mice with cardiac-specific overexpression of HMGB1 have shown that the protein enhances angiogenesis, restores cardiac function, and improves survival after myocardial infarction (MI) [112]. Autophagy inhibition in the heart, similar to that observed upon macrophage-stimulating 1 (Mst1, a proapoptotic kinase) activation, has been associated with p62 accumulation and aggregate formation accompanied by the disappearance of autophagosomes and cardiac dysfunction in mice subjected to MI [113].

As introduced previously, a fine balance between all mitochondrial quality control mechanisms is required for cell homeostasis. In IHD, a prominent role is played by mitochondrial biogenesis, the impairment of which is responsible for the altered energy production in the heart [114]. If mitophagy can be positively modulated (as described in Section 4) to rescue the phenotype of cardiomyocytes, peroxisome-proliferator-activated receptor coactivator (PGC)-1α may be strongly reactivated upon long-term exercise training to restore metabolism, although only partially [114].

### 2.3. Cardiomyopathies

Cardiomyopathy (CM) is a muscle-specific disorder of the heart characterized by abnormal myocardial structure and function occurring independently of any demonstrable CVDs, such as coronary artery disease (CAD), uncontrolled hypertension (HT), significant valvular heart disease, and congenital heart disorders. The prevalence of CM is 3% in the global population and increases to 12% in diabetic patients, leading to increased risks of HF and mortality [115]. Hyperglycemia, systemic and cardiac insulin resistance, increased free fatty acid (FFA) levels, renin-angiotensin-aldosterone system (RAAS) activation, sympathetic dysfunction, myocardial inflammation, oxidative stress, remodeling and fibrosis are factors that contribute to the development of CM. However, the exact pathophysiological mechanisms remain unclear [116].

Diabetic hearts have decreased rates of glucose oxidation and increased rates of FAO, resulting in oxidative stress, impaired OXPHOS, and eventually mitochondrial dysfunction. The increased FAO capacity is energetically detrimental and is mediated by the enhanced activity of peroxisome proliferator-activated receptor (PPAR)-α [117,118]. Additionally, metabolic stress-induced mitochondrial dysfunction, enhanced ROS production and increased Ca^2+^ overload-induced PTPC opening result in cardiomyocyte necrosis. Although increasing evidence has indicated that mitochondria are pivotal players in the pathophysiology and development of CM, the mechanism still remains to be investigated [119].

Efficient clearance of dysfunctional mitochondria through mitophagy is crucial for mitochondrial quality control and thus for the maintenance of cardiomyocyte viability [120]. Alterations in both autophagy and mitophagy have been extensively documented to be involved in the pathogenesis of several CMs [121]. Notably, several studies have revealed that the process of autophagy is inhibited in the hearts of type 1 and 2 diabetic mice, suggesting that inhibition of this process may contribute to diabetic CM (DCM) [122,123,124]. Interestingly, PINK1 and Parkin protein levels are significantly lower in type 1 diabetic hearts than in healthy hearts, leading to decreased cardiac mitophagy; this decrease in cardiac mitophagy is correlated with reduced expression of the small GTPase Rab9, which is involved in a noncanonical alternative selective autophagic pathway and is responsible for mitochondrial degradation during erythrocyte maturation in the absence of ATG5 [124,125,126]. Recent evidence has shown that mitochondrial dynamics play essential roles in the formation and maintenance of cardiomyocytes [127]. One role is linked to the importance of Drp1, the small GTPase involved in mitochondrial fission at the OMM, which is crucial for cardiac function and in response to energy stress [59,127,128,129]. Indeed, Drp1 deletion in cardiomyocytes results in decreased mitophagy and cardiac dysfunction, enhancing the risk of IRI [65,128].

Cahill TJ et al. reported that a single C452F mutation in Drp1 causes spontaneous development of monogenic dilated CM along with a wide variety of abnormal mitochondrial defects, including defective mitophagy [59] (Table 1). This missense mutation has been described as an enhancer of Drp1 GTPase activity that fails to guarantee the disassembly of the protein after oligomerization, thus causing it to become a partially dysfunctional protein. In the so-called Python heart, energetic failure may be ascribed to reduced mitochondrial Ca^2+^ uptake (as a consequence of ATPase Sarcoplasmic/Endoplasmic Reticulum Ca2+ Transporting 2 (SERCA2) inactivation) and to elevated numbers of inefficient mitochondria [59]. However, interruption of mitochondrial fusion is also related to rapid dilated CM onset, as observed in the adult heart upon ablation of both mitofusin 1 (MFN1) and 2 [130]. This deleterious effect is mainly attributable to a lack of fusion rather than to mitophagy dysfunction. In addition, mice bearing heart-specific deletion of either Mfn2 [131] or Park2 with concomitant expression of one Mfn2 mutant (a nonphosphorylatable form) [132] are predisposed to perinatal progressive CM and death within a few weeks after birth.

Many other studies on the importance of mitophagy in CM are present in the literature. For example, mitophagy can be considered a valid therapeutic target for transferrin receptor (*Tfrc*)-dependent CM, in which mice lacking this receptor develop lethal CM and drastically ineffective mitophagy [60]. During mitophagy, mtDNA is also degraded by DNase II in lysosomes. Incomplete digestion of mtDNA stimulates inflammation in cardiac tissue and is able to cause HF [133]. Recently, Tong M and associates demonstrated that during high-fat diet consumption, suppression of mitophagy increases the accumulation of lipids in the heart. The same study revealed that enhancement of mitophagy by Trans-Activator of Transcription (TAT)-Beclin1 tends to inhibit the development of CM [61] (Table 1). In a recent investigation, Wang S. et al. reported that mitophagy could be a target of melatonin treatment. Indeed, melatonin inhibits Mst1 phosphorylation, increases LC3-II levels and enhances Parkin-mediated mitochondrial removal to counteract the negative effects of CM [63]. In line with these observations, chronic treatment with metformin (classified as an antidiabetic and a potent inducer of autophagy), heme oxygenase-1 (HO-1) or mitochondrial aldehyde dehydrogenase (ALDH2) prevents CM by activating AMPK, normalizing cardiac autophagic activity, and inhibiting cardiomyocyte apoptosis through stimulation of Mitogen-Activated Protein Kinase 8 (JNK)-Bcl-2 signaling, thereby promoting the disruption of the Bcl-2-Beclin1 complex in diabetic heart tissue [123,134,135]. An additional downstream event is downregulation of nucleotide-binding domain, leucine-rich-containing family, pyrin domain-containing-3 (NLRP3) inflammasome levels in diabetic mice [136]. Interestingly, the NLRP3 inflammasome is considered a novel molecular marker in DCM that is activated by high FFA levels, hyperglycemia, and impaired insulin metabolic signaling.

Activation of the RAAS is considered a primary event in the etiology of both diabetes and HT (see below). RAAS activation contributes to insulin resistance in humans and correlates with increased levels of oxidative stress and cell death in affected hearts. Consistent with these effects and associations, the RAAS has been found to be upregulated during the pathogenesis of DCM. However, insulin signaling seems to have an additional role, especially at birth: inhibiting autophagy when a newborn starts to feed. Indeed, deletion of insulin receptor genes induces excessive organelle removal, contributing to myocyte loss and HF [137]. Overall, these findings support the importance of auto/mitophagy in ensuring the existence of a functional network of mitochondria and suggest that they provide cardioprotection.

### 2.4. Heart Failure

The 2016 European Society of Cardiology (ESC) guidelines for the diagnosis and treatment of acute and chronic HF state that “HF is a clinical syndrome characterized by typical symptoms (i.e., breathlessness, ankle swelling and fatigue) that may be accompanied by signs (i.e., elevated jugular venous pressure, pulmonary crackles and peripheral oedema) caused by a structural and/or functional cardiac abnormality, resulting in a reduced cardiac output and/or elevated intracardiac pressures at rest or during stress” [138].

This clinical scenario is strictly associated with mitochondrial function. To date, few studies have focused on the specific role of mitophagy in the heart, but emerging evidence supports a protective action for mitophagy in response to stress. Cardiomyocytes have been demonstrated to undergo caspase-independent cell death in the context of HF, and apoptosis, oncosis, and autophagy act simultaneously but play different roles in HF in humans [139,140].

In detail, failing hearts present impaired morphology of mitochondrial cristae, with disorganization and reduced cristae density [141]. These structural changes have also been detected in the hearts of Parkin-deficient mice, which exhibit disorganized mitochondrial networks and significantly reduced mitochondrial sizes; these mice are more sensitive to MI than their wild-type littermates, developing larger infarcts and exhibiting reduced survival. Parkin-KO cardiomyocytes present reduced mitophagy linked to dysfunctional mitochondria after MI [62] (Table 1). Moreover, evidence from ATG5-deficient mice has indicated that constitutive autophagy in the normal heart is a homeostatic mechanism for maintaining cardiomyocyte size and global cardiac structure and function, while upregulation of autophagy under HF conditions is an adaptive response to protect the heart from hemodynamic stress [142].

As introduced in the first section and as remarked upon previously, BNIP3 is a mediator of mitophagy and is activated during hypoxia; its effects have also been observed in an in vivo model of chronic HF [64]. In myocytes, mitophagy is a protective response in which Drp1-mediated mitochondrial fission and the recruitment of Parkin collaborate [143]. If disruption of Drp1 occurs, mitochondrial elongation increases with concomitant inhibition of mitophagy, promoting cardiac dysfunction and increased susceptibility to I/R. Similar evidence has also been obtained with tamoxifen-inducible cardiac-specific Drp1-KO mice [65]. In pig hearts, repetitive myocardial stunning induces autophagy as a homeostatic mechanism in which apoptosis is inhibited and tissue damage is limited. In fact, in this model, heart function fully recovers after normalization of coronary flow, suggesting that autophagy may promote the survival of hibernating myocardium [144].

A recent study has also suggested a role of mitophagy in HF pathogenesis (Figure 2). By using heart samples from HF patients and AMPKα2 genetically modified mouse models with transverse aortic constriction (TAC), researchers have found a serine residue (S495) in PINK1 that undergoes phosphorylation after exposure to AMPKα2 upon dissipation of the mitochondrial membrane potential in cardiomyocytes. This process has been found to be essential for the efficient translocation of Parkin to the mitochondria to increase the rate of mitophagy during HF [66] (Table 1). Moreover, PINK1 protein levels are markedly reduced in cases of end-stage human HF, indicating inefficient mitophagy [145].

In 2013, Hoshino A demonstrated that the impairment of mitophagy by cytosolic p53 facilitates HF in mice through binding of p53 to Parkin and subsequent p53 sequestration. This process impairs the reuse of dysfunctional mitochondria and subsequently induces the development of cardiac dysfunction [67] (Table 1). Recent studies on KO mouse models have also highlighted a role for Ulk1-dependent mitophagy in the protection of the heart against pressure overload-induced HF [146].

Coupled with impaired mitophagy, defects in mitochondrial biogenesis (and thus mtDNA depletion) occur due to downregulation of PGC-1α in the early stages of HF [147]. Moreover, failing hearts have been found to express depleted levels of nuclear respiratory factor 1 (NRF1), mitochondrial transcription factor A (TFAM) and estrogen-related receptor α (ERRα), and this reduction in expression has been demonstrated to negatively impact disease progression [148].

Most of the literature considers autophagy and mitophagy to be mediators of cardioprotection, especially when mitophagy removes damaged mitochondria; however, paradoxically, as observed in other heart diseases, several reports have described these pathways as degenerative processes in the context of HF, since their activation triggers a switch from compensated cardiac hypertrophy to HF with fibrosis onset [149]. In addition, stress-induced mitophagy in the failing heart is similar to a maladaptive response to hemodynamic parameters such as pressure overload and contributes to negative remodeling of the myocardium [150]. This process is mainly mediated by Beclin1 expression, as heterozygous disruption of Beclin1 in transgenic mice diminishes pathological remodeling.

### 2.5. Hypertension

HT is a multifactorial disease resulting from multiple causes and mechanisms controlling blood pressure, as Dr. Irvine H. proposed in a theory named the “mosaic of arterial hypertension” in 1967 [151]. Addressing HT is crucial because it remains a pivotal risk factor for CVDs.

Few studies have focused on mitophagy behavior in hypertensive hearts. In 2015, a study indicated that the coexistence of obesity and HT aggravates mitochondrial dysfunction, worsening left ventricular ejection fraction and general clinical outcomes in young animals. In this context, RAAS activation plays a dual role: it inhibits mitochondrial biogenesis by decreasing PGC-1α, NRF1 and TFAM and mediates the excessive clearance of mitochondria via mitophagy. The latter may occur following drastic changes in mitochondrial fission/fusion balance, where the cleavage of OPA1 (increased ratio of short isoform to long isoform) and the concomitant downregulation of MFN1 enhance mitochondrial fragmentation. Consequently, these dramatic modifications in mitochondrial turnover aggravate cell injury [68]. Taken together, these findings suggest that mitophagy may contribute to the development of hypertensive hearts, and further studies may provide new insights into these mechanisms. Further evidence derives from a murine model of HT induced by feeding mice a high-salt diet [69]. Spermidine assumption led to a delayed onset of HT in wild-type animals by stimulating autophagy and mitophagy in cardiomyocytes; however, spermidine had no beneficial effects in ATG5 KO mice, which lacked LC3-II and expressed high levels of p62/ Sequestosome 1 (SQSTM1) [69] (Table 1).

In addition to defects in this mitochondrial quality control system, general organelle dysfunction has been reported to be involved during HT [70,152,153]. At the level of the microvasculature of HT patients, capillary rarefaction is an important clinical sign to be evaluated. Capillary rarefaction may be counteracted by the action of endothelial progenitor cells (EPCs) strongly involved in angiogenesis, but mitochondrial dysfunction in these cells leads to a significant imbalance between microvasculature resistance and new vessel formation. Downregulation of the C-X-C motif chemokine receptor 4 (CXCR4)/Janus kinase 2 (JAK2)/sirtuin 5 (SIRT5) signaling pathway is responsible for EPC dysfunction exacerbating HT in patients; indeed, CXCR4, which is depleted in HT samples, is able to improve SIRT5-dependent mitochondrial function via JAK2 phosphorylation [70] (Table 1).

We aimed to provide a general overview of mitophagy/autophagy in HT diseases; however, we found conflicting evidence when considering pulmonary HT (PH), as further summarized in [153]. Briefly, increased levels of LC3-II were detected in almost all disease models (human, rat, mouse). The role of this increase is controversial, and the upstream (or downstream) mechanism remains unknown. In two cases, autophagy was considered cardioprotective by reducing vascular remodeling [154,155]. On the basis of other experimental data, autophagy was classified as detrimental since its ablation promoted angiogenesis and attenuated HF and HT [156,157].

### 2.6. Arrhythmia

Arrhythmia is a condition in which electric signals that allow the heart to beat do not work properly. Since electrical propagation supports proper cardiac function, arrhythmia can be fatal in some circumstances [158]. Thus far, there is no direct evidence linking defective mitophagy to the onset of this pathology, but mitochondria play key functional roles that depend on the amounts of ATP and ROS produced. Indeed, decreased ATP content and increased ROS generation negatively affect the electrical conduction of the heart through intracellular ion balance disruption [159], introducing heterogeneity in cardiac action potentials. The first link between mitophagy and proarrhythmic disturbances was revealed in 2019 in a study by Murphy KR and colleagues, who found that decreases in mitophagy due to aging lead to mitochondrial depolarization, ROS overproduction and consequent ryanodine receptor 2 (RyR2) oxidation; these changes induce altered and increased spontaneous Ca^2+^ waves (SCWs), enhancing the probability of arrhythmia [71] (Table 1). These findings have been further confirmed by the findings that ATG7 overexpression and Torin1 treatment restored mitochondrial polarization, normal ROS production and normal SCWs. For this reason, proper activation of the mitophagic (or autophagic) pathway could eliminate damaged organelles by preserving cell homeostasis.

### 2.7. Congenital Heart Disease

The incidence of moderate-to-severe structural congenital heart disease (CHD) in liveborn infants is 6 to 8 per 1000 live births; 3 per 1000 live births have heart disease that results in death or requires cardiac catheterization or surgery during the first year of life.

Recently, some authors [160,161] have found that anomalies in cilia function are implicated in CHDs. Cilia are hair-like organelles that can be found on the surfaces of different types of cells. They perform various functions, such as functions related to cell signaling, extracellular fluid propulsion and cell cycle control. The literature reports that motile and immotile cilia are required to establish left-right identity in developing embryos. During heart development, the most important role for cilia is establishing left-right asymmetry and determining the direction of heart looping [161]. Burkhalter MD reported a link between cilia and mitochondrial function during the development of heterotaxy syndromes. Impairment of mitochondrial function results in longer cilia, while shorter cilia are generated when organelle activity is enhanced. Interestingly, he also found a significant reduction in mtDNA in a cohort of heterotaxy patients [160]. Some important reports have provided proof of an association between autophagy and cilia length; in detail, cilia elongation depends on mTOR pathway suppression and thus autophagy activation, which enables suppression of proteasomal degradation of the proteins that constitute cilia [162,163,164].

Since autophagy is known to be involved in cardiomyocyte differentiation [165], impairment of this process may be the cause of some CHDs.

### 2.8. Peripheral Vascular Disease

Peripheral vascular disease (PVD) is a disorder of arteries and veins that causes pain and fatigue, often during physical exercise; however, damage to the vessel structure does not necessarily occur. VSMCs are the primary constituents of blood vessels, and they play very important roles, of which their capacity to transform into macrophage-like and osteochondrogenic-like cells is especially important. Several lines of evidence support the hypothesis that genetic reprogramming in VSMCs occurs when peripheral vessels become damaged [166]. VSMCs are currently believed to be exposed to autophagic stimuli such as oxidants, cytokines, ox-LDL and growth factors during PVD (i.e., after percutaneous coronary intervention) and that this molecular cascade determines the conversion of these cells to a macrophage-like phenotype, while the osteochondrogenic type is suppressed [167]. Thus, autophagy appears to be a regulator of the cell response to injury. In the most common PVD, deep venous thrombosis (DVT), ATG5 plays a critical role in the progression of the pathology since it mediates thrombus recanalization, acting mainly on endothelial cells. In this context, ATG5 activates Akt phosphorylation, which is counteracted by the use of 3-methyladenine (3-MA); 3-MA inhibits autophagy by lowering ATG5 levels and concomitantly inhibiting endothelial cell migration and tube formation [168]. Strong evidence of the role of autophagy in promoting angiogenesis has also been derived from other studies on pathologies requiring considerable tissue-remodeling programs, such as cancers, and from findings following the pharmacological inhibition of this pathway. Indeed, the enhanced presence of lysosome-associated membrane protein (LAMP) and cathepsin D drives robust angiogenesis in the context of infantile hemangioma [169,170]. Otherwise, chronic expression of ATG5 induces cell death.

Patients with peripheral artery disease usually present with decreased mobility, which cannot be explained only by ischemia-related events or pain. Many reports that have focused on muscle fiber types and their distributions in PVD patients compared to non-PVD participants have reported impaired mitochondrial respiration, enhanced oxidative stress and loss of intermyofibrillar mitochondria [72]. In addition, excessive mitophagy has been detected in parallel with the loss of compensatory mechanisms, such as PGC-1α overexpression. This uncontrolled and dysfunctional mitochondrial removal is independent of age and has been evaluated by direct assessment of LC3-II increases in fibers [72] (Table 1). Although further experiments are needed to fully address the causes and consequences associated with mitophagy, the findings thus far may help to guide research towards new treatments for this group of diseases. For further details, please also refer to [121].

### 2.9. Mitophagy in Human Cardiac Surgery

Nevertheless, many people are subjected to cardiac surgery involving coronary artery bypass grafting (CABG), and despite the use of cardioplegic solutions to preserve heart function, I/R remains one of the main challenges in this periprocedural setting.

In 2013, a descriptive paper reported that the heart of patients undergoing CABG activates the homeostatic intracellular repair response (HIR) program, a prevailing aspect of which is autophagy activation [171]. In heart samples from 19 patients, p62, Beclin1, ATG5 and LC3-II degradation were recorded, suggesting an increased autophagic flux [171]. The same authors later asserted that mitophagy and mitochondrial biogenesis also take part in the HIR program by increasing their activation following cardiac surgery [73]. Despite the innovative approach and use of human samples in these studies, additional studies should further confirm this evidence because autophagy and mitophagy are complex and difficult to assess in tissues. For example, the low levels of LC3-II detected in the first study [171] indicate both low autophagy induction and increased flux without protein replacement. In the second paper [73], mitophagy activation was inferred from Parkin translocation from the cytosol to mitochondria in heart samples before and after surgery and from decreased levels in optineurin (OPTN) and NDP52 receptors in the total homogenate; however, the translocation of these proteins into mitochondria and their clustering should be evaluated. Notably, a high percentage of patients being analyzed might be under statin treatment, which induces autophagy itself [172].

Nevertheless, mitophagy activation during CABG, assessed by FUNDC1 dephosphorylation, has also been reported by a second independent group, but this was described as a deleterious event in the follow-up of patients as it caused platelet aggregation and increased risk of thrombosis [74].

## 3. Targeting Mitophagy for Cardioprotection

As a mechanism critical for mitochondrial quality control, mitophagy is essential for the establishment of a healthy mitochondrial population inside cells. We have discussed how disruption of mitophagic and autophagic pathways exacerbates the development of heart diseases, but it is important to understand how these pathways modulate heart diseases for therapeutic purposes.

Various chemical reagents, drugs and natural compounds are able to modulate mitophagy (Table 2), and studies on their use in therapies are in progress.

Polyphenols are plant-derived compounds that offer many health benefits. Recent studies have revealed the ability of these compounds to alter mitophagy via different signaling pathways; for example, resveratrol and quercetin act on FOXO3a signaling and promote PINK1 and Parkin overexpression [173], curcumin regulates transcription factor EB (TFEB) activity via the inhibition of mTORC1 [174], and melanoidin activates mitophagy by increasing the levels of Beclin1 [175]. The mTOR pathway is also the target of a drug belonging to the statin class, simvastatin, which inhibits cholesterol biosynthesis. Simvastatin acts on the mitochondrial translocation of Parkin and p62/SQSTM1 to increase mitophagy and autophagy [172], which is required for cardioprotection.

On the other hand, also excessive and persistent mitophagy can also be deleterious; in this context, Parkin, PINK1 and Beclin1 proteins may be upregulated. This upregulation can be counteracted by multiple types of treatments, such as those involving the use of the superoxide dismutase (SOD) mimetic 4-hydroxy-2,2,6,6-tetramethylpiperidin-1-oxyl (TEMPOL) and mitoTEMPOL [176,177], which counteract age-related mitophagy impairment associated with diseases. Other modulators of mitophagy mediators are zinc [178] and liraglutide [179]. Zinc is able to ensure proper mitophagic pathway signaling through PINK1 and Beclin1 by limiting ROS generation and mitochondrial membrane potential dissipation, while liraglutide, an agonist of the glucagon-like peptide 1 (GLP1) receptor, upregulates the sirtuin 1 (SIRT1) pathway, promoting Parkin-mediated mitophagy, and has already been validated as a molecule useful for the repair of infarcted hearts. Another SIRT1 activator with similar effects related to mitophagy and cardioprotection is nicotinamide [180].

Among all the antioxidants mentioned so far, melatonin plays a very important role in cardioprotection. Although melatonin is known to be a master regulator of cell death and inflammation [181], a few studies have reported its roles in inducing autophagy [182,183] and mitophagy during CVDs. In addition to the two studies mentioned in the “Atherosclerosis” and “Cardiomyopathies” sections [53,63], other studies have been conducted on ischemia and reperfusion conditions, during which mitochondrial fission is activated and the PTPC is in its open state [184]. Another study reported upregulation of the PINK1/Parkin pathway and cell death mediated by mitophagy [185]. These (perhaps apparently) controversial results require further research in the field to clearly evaluate the molecular mechanisms associated with cardioprotection. Similarly, the link between PTPC activity and mitophagy/autophagy has not yet been fully addressed [91,186]. Mitochondrial dynamics are strongly linked to mitophagy; consequently, therapies targeting proteins involved in fission or fusion may also act on mitophagy. Mfn2, which is essential for mitochondrial fusion, has also been found to be a receptor for Parkin recruitment to mitochondria that should undergo mitophagy [131]; this process is regulated by PINK1 phosphorylation involving the T111/S442 residues, the simultaneous mutation of which prevents functional interactions between Mfn2 and Parkin. The absence of Mfn2 prevented Parkin translocation and thus mitophagy [131]. Mfn2 mutations are reportedly able to cause diseases such as the neurodegenerative Charcot Marie Tooth type 2A, and the use of agonists to improve Mfn2 actions reverses mitochondrial dysfunction [187]. Mfn2 can also be phosphorylated at S378 by PINK1, and this phosphorylation site is essential to its functions. Therefore, Mfn2 agonists may also act on mitophagy as well as mitochondrial fusion to counteract the negative effects of the disease.

## 4. Future Perspectives and Conclusions

The crucial role of mitochondria in cardiac function is well established; thus, a fine balance between the removal of dysfunctional mitochondria and their replacement with new organelles is needed. Overall, induction of mitophagy is seen as a cardioprotective mechanism started by the heart to protect itself from different types of damage, but to what extent (coupled to the setting) mitophagy may be beneficial and the circumstances in which it can be considered detrimental are not yet clear. In addition, the heart is characterized by three different mitochondrial subpopulations, and it would be interesting to understand whether there are differences in mitophagy among these subpopulations and the purpose of these differences. Therefore, a threefold evaluation of mitochondrial dynamics, biogenesis and PTPC opening should be considered when examining mitophagy.

The proper use of disease animal models combined with cardiac-specific deletion of proteins involved in such pathways and in-depth analysis of human samples may provide further insights to address conflicting evidence and unanswered questions. Recent important findings highlight the role of microRNA (miR)-dependent regulation of gene expression in cardiac protection and repair [188]. An interesting association between miRs and mitophagy has also been reported. Under experimental I/R conditions, upregulation of miR-410 and concomitant loss of mitophagy have been found to occur [189]; under these circumstances, miR-410 was associated with decreased cell viability and severe mitochondrial impairment, which may occur via direct interaction with HMGB1 and subsequent mitophagy inhibition. A second miR (miR-137) has also been detected to be overexpressed under the same conditions; this miR is able to downregulate Nix and FUNDC1, impairing mitophagy [190]. Therefore, controlling and monitoring the expression of miRs may be an added value for cardioprotective therapies.

## Figures and Tables

**Table 1:** Summary of literature describing mitophagic pathways in cardiovascular diseases.

Pathology	Mechanism of Action	References
Atherosclerosis	Atherosclerotic plaque stabilization mediated by ox-LDL.	[52]
	NLRP3 inflammasome activation by the SIRT3/FOXO3a/Parkin signaling pathway.	[53]
Ischemic heart disease	Mitophagy is an adaptive metabolic response to hypoxia mediated by BNIP3, Beclin-1 and ATG5.	[54]
	Pigment epithelium-derived factor (PEDF) protects hypoxic cardiomyocytes in a rat model through the PEDF/PEDF-R/PA/DAG/PKC-α/ULK1/FUNDC1 pathway.	[55]
	*Fundc1*-knockout (KO) platelets present impaired mitochondria, which cause more I/R heart injury.	[56]
	FUNDC1 loss of function through CK2α-mediated phosphorylation leads to the development of cardiac I/R injury in mice.	[57]
	HMGB1 treatment in a murine model inhibits apoptosis via mTORC1 inhibition.	[58]
Cardiomyopathies and heart failure	Inhibition of the dynamic process through the expression of dominant-negative Drp1 (Drp1^K38A^) results in disruption of mitophagy. C452F Drp1 mutation causes spontaneous development of monogenic dilated CM.	[59]
	Mice lacking transferrin receptor (Tfrc) develop lethal CM and drastically ineffective mitophagy due to altered expression of proteins involved in mitophagy.	[60]
	Enhancement of mitophagy by TAT-Beclin1 attenuates the development of DCM.	[61]
	Melatonin inhibits Mst1 phosphorylation, increases LC3-II levels and enhances Parkin activity in mice with CM.	[62]
	Parkin-deficient mice are more sensitive to MI, developing larger infarcts and exhibiting reduced survival.	[63]
	An increase in BNIP3 expression is detected in adult rat hearts with chronic HF.	[64]
	Tamoxifen-inducible cardiac-specific Drp1-KO mice present cardiac dysfunction and increased susceptibility to I/R linked to an accumulation of damaged mitochondria (due to mitophagy inhibition).	[65]
	Phosphorylation of Ser495 in PINK1 by AMPKα2 prevents the progression of HF.	[66]
	Cytosolic p53 binding to Parkin prevents its translocation to damaged mitochondria, modulating cardiac dysfunction in HF.	[67]
Hypertension	The coexistence of obesity and HT aggravates mitochondrial dysfunction; RAAS activation inhibits mitochondrial biogenesis.	[68]
	Spermidine assumption leads to a delayed onset of HT in wild-type animals by stimulating mitophagy in cardiomyocytes, but these effects are abolished in ATG5-KO mice.	[69]
	EPCs in HT patients present mitochondrial dysfunctions linked to impairment of the CXCR4/JAK2/SIRT5 signaling pathway and failure of angiogenic capacity.	[70]
Arrhythmia	Decrease in mitophagy leads to pro-arrhythmic spontaneous Ca^2+^ release via oxidized RyR2s by mito-ROS.	[71]
Peripheral vascular disease	PVD fibers present an accumulation of LC3 that is not completely colocalized with LAMP2.	[72]
Mitophagy in cardiac surgery	Post-cardiopulmonary bypass samples display decreased levels of mitophagy adapters NDP52 and OPTN, decreased expression of the long form of Opa1, and translocation of Parkin to the mitochondrial fraction.	[73]
	Reperfusion after CABG surgery induces FUNDC1 dephosphorylation and mitophagy activation, causing platelet aggregation and increased risk of thrombosis.	[74]

Ox-LDL: oxidized low-density lipoprotein; NLRP3: Nucleotide-binding domain, leucine-rich-containing family, pyrin domain-containing-3; SIRT3: Sirtuin 3; FOXO3a: Forkhead Box O3; Parkin: Parkin RBR E3 Ubiquitin Protein Ligase; BNIP3: BCL2 Interacting Protein 3; ATG5: Autophagy related 5; PEDF: Pigment epithelial-derived factor; PEDF-R: PEDF receptor; PA: palmitic acid; DAG: diacylglycerol; PKC-α: protein kinase C alpha; ULK1: Unc-51 Like Autophagy Activating Kinase 1; FUNDC1: FUN14 Domain Containing 1; CK2α: protein kinase CK2 alpha; I/R: ischemia/reperfusion; HMGB1: High Mobility Group Box 1; mTORC1: mammalian target of rapamycin complex 1; CM: cardiomyopathy; TAT_Beclin1: Trans-Activator of Transcription-Beclin1; DCM: diabetic cardiomyopathy; LC3-II: Microtubule Associated Protein 1 Light Chain 3 II; MI: myocardial infarction; HF: heart failure; PINK1: PTEN Induced Kinase 1; AMPKα2: adenosine monophosphate protein kinase alpha 2; RAAS: renin–angiotensin–aldosterone system; ATG5-KO: autophagy related 5-knock out; CXCR4: C-X-C Motif Chemokine Receptor 4; JAK2: Janus Kinase 2; SIRT5: Sirtuin 5; RYR2: Ryanodine Receptor 2; mito-ROS: mitochondrial reactive oxygen species; PVD: peripheral vascular disease; LAMP2: Lysosomal Associated Membrane Protein 2; NDP52: Nuclear Domain 10 Protein 52; OPTN: optineurin; CABG: Coronary Artery Bypass Graft.

**Table 2:** Summary of compounds that provide significant cardioprotection via mitophagy regulation.

Compound	Target/Mechanism of Action	Cardiovascular Effect
TEMPOL	SOD mimetic/reduces ROS and counteracts age-related mitophagy	Improves preconditioning after hypoxia/reoxygenation in aged cultured cardiomyocytes
Simvastatin	mTOR-dependent activation of mitophagy through Parkin and p62/SQSTM1	Improves cardioprotection during left ventricular artery occlusion and reperfusion
Zinc	Favors SOD activity and activates PINK1/Beclin1-dependent mitophagy	Improves the recovery of cultured cardiomyocytes after hypoxia/reoxygenation
Liraglutide	Agonist of GLP1, stimulates SIRT1 and promotes Parkin-mediated mitophagy	Promotes recovery after left ventricular artery ligation-induced ischemia
Melatonin	Inhibits Mst1 phosphorylation, increases LC3-II levels and enhances Parkin-mediated mitophagy	Counteracts diabetic CM
Metformin	Activates autophagy by disrupting the Bcl2-Beclin1 complex	Inhibits cardiomyocyte apoptosis in diabetic heart

TEMPOL: 4-hydroxy-2,2,6,6-tetramethylpiperidin-1-oxyl; SOD: superoxide dismutase 1; ROS: reactive oxygen species; SQSTM1: Sequestosome 1; PINK1: PTEN Induced Kinase 1; GLP1: Glucagon Like Peptide 1; SIRT1: Sirtuin 1; LC3-II: Microtubule Associated Protein 1 Light Chain 3 II; CM: cardiomyopathy.
